# Supplementary material for: Ferritin and C-reactive protein are predictive biomarkers of mortality and macrophage activation syndrome in adult onset Still’s disease. Analysis of the multicentre Gruppo Italiano di Ricerca in Reumatologia Clinica e Sperimentale (GIRRCS) cohort
Source: PLoS One. 2020 Jul 9;15(7):e0235326. doi: 10.1371/journal.pone.0235326 (PMC7347102; doi:10.1371/journal.pone.0235326)
Supplement: S2 Table — (DOC) [file pone.0235326.s002.doc]

**S2 Table. Univariate regression analyses assessing possible clinical predictors of MAS**

| MAS | OR | SE | P | CI 95% |
| --- | --- | --- | --- | --- |
| **Univariate analyses** | | | | |
| Age | 1.016 | 0.013 | 0.253 | 0.989-1.043 |
| Gender | 1.585 | 0.463 | 0.320 | 0.639-3.929 |
| Arthritis | 0.662 | 0.618 | 0.504 | 0.197-2.222 |
| Skin Rash | 0.895 | 0.490 | 0.820 | 0.343-2.337 |
| Splenomegaly | 2.400 | 0.532 | 0.705 | 0.846-6.812 |
| Myalgia | 3.616 | 0.574 | **0.025** | 1.173-11.149 |
| Liver involvement | 3.060 | 0.531 | **0.035** | 1.082-8.657 |
| Sore throat | 3.997 | 0.530 | **0.009** | 1.415-11.290 |
| Lymph node | 4.414 | 0.530 | **0.005** | 1.562-12.467 |
| Pericarditis | 2.976 | 0.469 | **0.020** | 1.187-7.465 |
| Pleuritis | 2.674 | 0.479 | **0.040** | 1.045-6.842 |
| Abdominal pain | 3.061 | 0.531 | **0.035** | 1.082-8.661 |
| AOSD pneumonia | 1.390 | 0.613 | 0.592 | 0.418-4.624 |
| Systemic Score | 1.618 | 0.120 | **0.0001** | 1.278-2.048 |
| Leucocytosis >15000mm^3^ | 1.255 | 0.437 | 0.602 | 0.533-2.955 |
| Ferritin | 1.945 | 0.209 | **0.001** | 1.292-2.928 |
| ESR | 1.012 | 0.008 | 0.152 | 0.996-1.028 |
| CRP | 1.279 | 0.192 | 0.199 | 0.878-1.862 |
| Low dosage of CCSs | 0.360 | 0.500 | 0.541 | 0.135-1.959 |
| sDMARDs | 2.044 | 0.502 | 0.354 | 0.765-5.466 |
| bDMARDs | 0.836 | 0.484 | 0.712 | 0.324-2.160 |
| Monocyclic pattern | 0.267 | 0.574 | 0.679 | 0.087-1.823 |
| Polycyclic pattern | 0.318 | 0.575 | 0.347 | 0.103-1.983 |
| Chronic pattern | 1.680 | 0.464 | 0.264 | 0.676-4.170 |

MAS=macrophage activation syndrome; AOSD=Adult Onset Still’s Disease; CCSs=Corticosteroids; ESR=Erythrocyte Sedimentation Rate; CRP=C Reactive Protein; sDMARDs= synthetic Disease Modifying Anti-Rheumatic Drugs; bDMARDs=biologic Disease Modifying Anti-Rheumatic Drugs; OR=odds ratio; SE=standard error; P=p-value; CI=confidence interval. Statistical significance was expressed by a p value <0.05. Bolded values indicate statistically significant results.
