# Supplementary material for: Ferritin and C-reactive protein are predictive biomarkers of mortality and macrophage activation syndrome in adult onset Still’s disease. Analysis of the multicentre Gruppo Italiano di Ricerca in Reumatologia Clinica e Sperimentale (GIRRCS) cohort
Source: PLoS One. 2020 Jul 9;15(7):e0235326. doi: 10.1371/journal.pone.0235326 (PMC7347102; doi:10.1371/journal.pone.0235326)
Supplement: S3 Table — (DOC) [file pone.0235326.s003.doc]

**S3 Table. Univariate regression analyses assessing possible clinical predictors of ferritin ≥ 1225 ng/ml**

| **FERRITIN ≥ 1225 ng/ml** | **OR** | **SE** | **P** | **CI 95%** |
| --- | --- | --- | --- | --- |
| **Univariate analyses** | | | | |
| Age | 1.014 | 0.010 | 0.190 | 0.993-1.035 |
| Gender | 0.933 | 0.344 | 0.839 | 0.475-1.831 |
| Arthritis | 0.437 | 0.561 | 0.140 | 0.145-1.312 |
| Skin Rash | 3.571 | 0.412 | **0.002** | 1.592-8.011 |
| Splenomegaly | 2.353 | 0.366 | **0.019** | 1.149-4.821 |
| Myalgia | 2.454 | 0.357 | **0.012** | 1.219-4.942 |
| Liver involvement | 2.588 | 0.354 | **0.007** | 1.294-5.178 |
| Sore throat | 1.140 | 0.337 | 0.697 | 0.589-2.209 |
| Lymph node | 2.427 | 0.343 | **0.010** | 1.238-4.755 |
| Pericarditis | 2.137 | 0.428 | 0.076 | 0.924-4.944 |
| Pleuritis | 3.429 | 0.472 | **0.009** | 1.358-8.654 |
| Abdominal pain | 1.074 | 0.484 | 0.883 | 0.416-2.774 |
| AOSD pneumonia | 7.863 | 0.773 | **0.008** | 1.727-35.803 |
| Leukocytosis >15000mm^3^ | 1.623 | 0.337 | 0.351 | 0.838-3.144 |
| Low dosage of CCSs | 0.440 | 0.347 | 0.718 | 0.223-1.867 |
| sDMARDs | 0.715 | 0.352 | 0.341 | 0.359-1.426 |
| bDMARDs | 0.550 | 0.366 | 0.102 | 0.269-1.127 |
| Monocyclic pattern | 1.093 | 0.350 | 0.799 | 0.551-2.170 |
| Polycyclic pattern | 0.672 | 0.357 | 0.266 | 0.334-1.353 |
| Chronic pattern | 1.103 | 0.380 | 0.796 | 0.524-2.322 |

AOSD=Adult Onset Still’s Disease; CCSs=Corticosteroids; ESR=Erythrocyte Sedimentation Rate; sDMARDs= synthetic Disease Modifying Anti-Rheumatic Drugs; bDMARDs=biologic Disease Modifying Anti-Rheumatic Drugs; N=Number; OR=odds ratio; SE=standard error; P=p-value; CI=confidence interval. Statistical significance was expressed by a p value <0.05. Bolded values indicate statistically significant results.
