# Supplementary material for: Ferritin and C-reactive protein are predictive biomarkers of mortality and macrophage activation syndrome in adult onset Still’s disease. Analysis of the multicentre Gruppo Italiano di Ricerca in Reumatologia Clinica e Sperimentale (GIRRCS) cohort
Source: PLoS One. 2020 Jul 9;15(7):e0235326. doi: 10.1371/journal.pone.0235326 (PMC7347102; doi:10.1371/journal.pone.0235326)
Supplement: S4 Table — (DOC) [file pone.0235326.s004.doc]

**S4 Table. Univariate regression analyses assessing possible clinical predictors of mortality**

| **MORTALITY** | **OR** | **SE** | **P** | **CI 95%** |
| --- | --- | --- | --- | --- |
| **Univariate analyses** | | | | |
| Age | 1.035 | 0.016 | **0.029** | 1.004-1.067 |
| Gender | 1.246 | 0.503 | 0.661 | 0.465-3.337 |
| Arthritis | 0.456 | 0.631 | 0.214 | 0.132-1.571 |
| Skin Rash | 0.753 | 0.530 | 0.593 | 0.267-2.129 |
| Splenomegaly | 1.194 | 0.523 | 0.734 | 0.429-3.328 |
| Myalgia | 5.844 | 0.767 | **0.021** | 1.299-26.287 |
| Liver involvement | 1.167 | 0.503 | 0.759 | 0.435-3.127 |
| Sore throat | 5.237 | 0.651 | **0.011** | 1.462-18.758 |
| Lymph node | 5.757 | 0.651 | **0.007** | 1.607-20.616 |
| Pericarditis | 1.749 | 0.537 | 0.298 | 0.611-5.005 |
| Pleuritis | 1.431 | 0.564 | 0.525 | 0.474-4.321 |
| Abdominal pain | 4.722 | 0.553 | **0.005** | 1.598-13.950 |
| AOSD pneumonia | 5.678 | 0.565 | **0.002** | 1.876-17.188 |
| Systemic Score | 1.492 | 0.124 | **0.001** | 1.171-1.901 |
| Leukocytosis >15000mm^3^ | 1.095 | 0.483 | 0.852 | 0.424-2.823 |
| Ferritin | 1.318 | 0.765 | 0.182 | 0.879-1.975 |
| ESR | 1.014 | 0.009 | 0.130 | 0.996-1.033 |
| CRP | 2.275 | 0.268 | **0.002** | 1.346-3.844 |
| Low dosage of CCSs | 0.210 | 0.651 | 0.617 | 0.059-1.752 |
| sDMARDs | 1.019 | 0.504 | 0.970 | 0.379-2.738 |
| bDMARDs | 3.481 | 0.493 | 0.611 | 0.325-9.148 |
| Monocyclic pattern | 1.000 | 5520.929 | 0.997 | 0.0001-0 |
| Polycyclic pattern | 1.000 | 5801.356 | 0.997 | 0.0001-0 |
| Chronic pattern | 1.000 | 6520.161 | 0.998 | 0.0001-0 |

AOSD=Adult Onset Still’s Disease; CCSs=Corticosteroids; ESR=Erythrocyte Sedimentation Rate; sDMARDs= synthetic Disease Modifying Anti-Rheumatic Drugs; bDMARDs=biologic Disease Modifying Anti-Rheumatic Drugs; OR=odds ratio; SE=standard error; P=p-value; CI=confidence interval. Statistical significance was expressed by a p value <0.05. Bolded values indicate statistically significant results.
