# Supplementary material for: Ferritin and C-reactive protein are predictive biomarkers of mortality and macrophage activation syndrome in adult onset Still’s disease. Analysis of the multicentre Gruppo Italiano di Ricerca in Reumatologia Clinica e Sperimentale (GIRRCS) cohort
Source: PLoS One. 2020 Jul 9;15(7):e0235326. doi: 10.1371/journal.pone.0235326 (PMC7347102; doi:10.1371/journal.pone.0235326)
Supplement: S5 Table — (DOC) [file pone.0235326.s005.doc]

**S5 Table. Univariate regression analyses assessing possible predictors of CRP ≥ 68.5 mg/L**

| **CRP ≥ 68.5 mg/L** | **OR** | **SE** | **P** | **CI 95%** |
| --- | --- | --- | --- | --- |
| **Univariate analyses** | | | | |
| Age | 1.019 | 0.011 | 0.084 | 0.998-1.040 |
| Gender | 1.698 | 0.348 | 0.129 | 0.858-3.361 |
| Arthritis | 0.612 | 0.518 | 0.343 | 0.222-1.688 |
| Skin Rash | 0.608 | 0.383 | 0.193 | 0.287-1.287 |
| Splenomegaly | 1.598 | 0.362 | 0.195 | 0.785-3.250 |
| Myalgia | 2.772 | 0.374 | **0.006** | 1.332-5.769 |
| Liver involvement | 1.212 | 0.346 | 0.578 | 0.616-2.387 |
| Sore throat | 0.797 | 0.337 | 0.499 | 0.412-1.541 |
| Lymph node | 2.045 | 0.343 | 0.037 | 1.044-4.007 |
| Pericarditis | 0.451 | 0.455 | **0.080** | 0.185-1.101 |
| Pleuritis | 0.399 | 0.451 | **0.042** | 0.165-0.966 |
| MAS | 1.469 | 0.434 | 0.375 | 0.628-3.438 |
| Abdominal pain | 1.442 | 0.482 | 0.448 | 0.560-3.711 |
| AOSD pneumonia | 1.851 | 0.507 | 0.225 | 0.685-5.001 |
| Leucocytosis >15000mm^3^ | 0.809 | 0.335 | 0.526 | 0.419-1.558 |
| Low dosage of CCSs | 0.309 | 0.363 | 0.671 | 0.152-3.629 |
| sDMARDs | 0.778 | 0.348 | 0.470 | 0.393-1.539 |
| bDMARDs | 2.347 | 0.367 | 0.520 | 0.143-4.820 |
| Monocyclic pattern | 0.663 | 0.353 | 0.245 | 0.332-1.325 |
| Polycyclic pattern | 0.658 | 0.363 | 0.249 | 0.323-1.341 |
| Chronic pattern | 0.545 | 0.398 | 0.127 | 0.249-1.189 |

CRP=C Reactive Protein; AOSD=Adult Onset Still’s Disease; CCSs=Corticosteroids; ESR=Erythrocyte Sedimentation Rate; sDMARDs= synthetic Disease Modifying Anti-Rheumatic Drugs; bDMARDs=biologic Disease Modifying Anti-Rheumatic Drugs; N=Number; OR=odds ratio SE=standard error; P=p-value; CI=confidence interval. Statistical significance was expressed by a p value <0.05. Bolded values indicate statistically significant results.
